# Anti-Inflammatory Mechanism of An Alkaloid Rutaecarpine in LTA-Stimulated RAW 264.7 Cells: Pivotal Role on NF-κB and ERK/p38 Signaling Molecules

**DOI:** 10.3390/ijms23115889

**Published:** 2022-05-24

**Authors:** Thanasekaran Jayakumar, Chun-Ming Yang, Ting-Lin Yen, Chia-Yuan Hsu, Joen-Rong Sheu, Chih-Wei Hsia, Manjunath Manubolu, Wei-Chieh Huang, Cheng-Ying Hsieh, Chih-Hsuan Hsia

**Affiliations:** 1 Graduate Institute of Medical Sciences, College of Medicine, Taipei Medical University, Taipei 110, Taiwan; jayakumar@tmu.edu.tw (T.J.); dr.yang1101@gmail.com (C.-M.Y.); m120107023@tmu.edu.tw (C.-Y.H.); sheujr@tmu.edu.tw (J.-R.S.); d119106003@tmu.edu.tw (C.-W.H.); d119110003@tmu.edu.tw (W.-C.H.); 2 Department of Neurology, Chi Mei Medical Center, Tainan 710, Taiwan; 3 Department of Medical Research, Cathay General Hospital, Taipei 106, Taiwan; d119096015@tmu.edu.tw; 4 Department of Pharmacology, School of Medicine, College of Medicine, Taipei Medical University, Taipei 110, Taiwan; 5 Department of Evolution, Ecology and Organismal Biology, Ohio State University, Columbus, OH 43212, USA; manubolu.1@osu.edu; 6 Translational Medicine Center, Shin Kong Wu Ho-Su Memorial Hospital, Taipei 111, Taiwan

**Keywords:** rutaecarpine, anti-inflammation, MAPK/NF-κB signaling pathways, inflammatory molecules

## Abstract

Lipoteichoic acid (LTA) is a key cell wall component and virulence factor of Gram-positive bacteria. LTA contributes a major role in infection and it mediates inflammatory responses in the host. Rutaecarpine, an indolopyridoquinazolinone alkaloid isolated from *Evodia rutaecarpa,* has shown a variety of fascinating biological properties such as anti-thrombotic, anticancer, anti-obesity and thermoregulatory, vasorelaxing activity. It has also potent effects on the cardiovascular and endocrine systems. Herein, we investigated rutaecarpine’s (Rut) anti-inflammatory effects in LTA-stimulated RAW macrophage cells. The Western blot and spectrophotometric results revealed that Rut inhibited the production of nitric oxide (NO) and the expression of inducible nitric oxide synthase (iNOS), cyclooxygenase-2 (COX-2), and interleukin (IL)-1β in the LTA-induced macrophage cells. Successively, our mechanistic studies publicized that Rut inhibited LTA-induced phosphorylation of mitogen-activated protein kinase (MAPK) including the extracellular signal-regulated kinase (ERK), and p38, but not c-Jun NH2-terminal kinase (JNK). In addition, the respective Western blot and confocal image analyses exhibited that Rut reserved nuclear transcription factor kappa-B (NF-κB) by hindering inhibitor of nuclear factor κB-α (IκBα) and NF-κB p65 phosphorylation and p65 nuclear translocation. These results indicate that Rut exhibits its anti-inflammatory effects mainly through attenuating NF-κB and ERK/p38 signaling pathways. Overall, this result suggests that Rut could be a potential therapeutic agent for the treatment of Gram-positive bacteria induced inflammatory diseases.

## 1. Introduction

Lipoteichoic acids (LTA), a major component of Gram-positive bacteria cell membrane, were publicized to be one of the serious factors contributing to the pathogenesis of sepsis [1]. This cell component can stimulate inflammatory responses in the lung [2]. Thus, understanding the mechanisms that control LTA-mediated cell activation is critical for diagnosis, treatment, or prognosis of lung inflammatory diseases. LTA can trigger macrophages to produce huge quantities of inflammatory factors that reveal systemic effects in the general circulation [3]. Various cytokines such as interleukin (IL)-1β, IL-6, and tumor necrosis factor (TNF)-α [4] have been reported to be induced by LTA stimulation. Mitogen-activated protein kinases (MAPKs) are serine/threonine kinases. The extracellular signal-regulated kinase 1 and 2 (ERK 1/2) were the first MAPK isoforms to be cloned and characterized [5]. Mitogen-activated/ERK kinases (MEKs) are a well-documented family of dual-specificity kinases that have been reported to activate ERK 1/2 [3,6]. An earlier study confirmed that LTA can particularly stimulate the ERK pathway in the cornea [7]. Another previous study displayed that LTA induces TNF-α and IL-6 expressions in macrophages via stimulating the phosphorylation of ERK1/2 [3].

Apart from the secretion of proinflammatory cytokines TNF, IL-1β, and IL-6, LTA augmented the production of nitric oxide (NO) [8] and cyclooxygenase (COX)-2, all of these molecules have been found to be induced through phosphorylation of nuclear factor-κB (NF-κB) and MAPK [9]. While bacterial cells undergo bacteriolysis induced by leukocyte lysozymes or β-lactam antibiotics, the cells release LTA and bind to target cells either non-specifically via membrane phospholipids, or specifically via CD14 and Toll-like receptor 2, ensuing in the release of reactive oxygen and nitrogen species, acid hydrolase, proteinase, bactericidal peptides, and proinflammatory cytokines [10]. Together, studies stated that LTA is linked to the pathophysiology of inflammation such as septic shock, adult respiratory distress syndrome, and toxic shock syndrome, therefore proposing that LTA is one of the major virulence factors of Gram-positive bacteria [10,11]. Overall, it is clearly confirmed that inhibition of LTA-induced inflammatory responses through MAPK and NF-κB pathways may offer potential therapeutic targets in inflammatory diseases.

**Figure 1:**
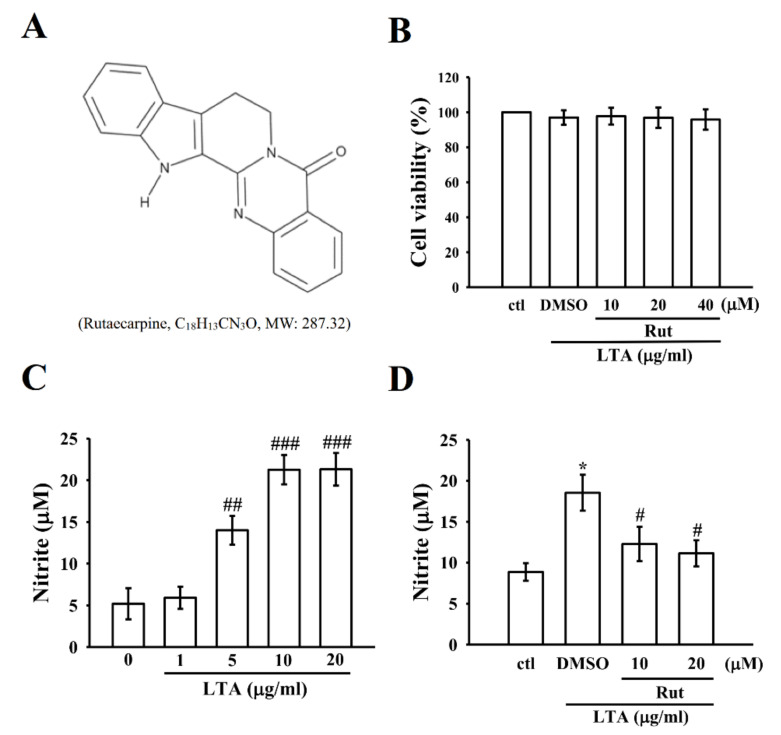
Effects of lipoteichoic acid (LTA) and rutaecarpine (Rut) on cell viability and nitric oxide (NO) production in RAW 264.7 cells. (**A**) Chemical structure of rutaecarpine. (**B**) Cells were treated with Rut (10–40 μM) for 20 min and then treated with LTA (10 μg/mL) for 24 h. Cell viability was assessed as described in the Methods section. Cells were treated with (**C**) various concentrations of LTA (1–20 μg/mL) for 24 h (**D**) 0.1% dimethyl sulfoxide (DMSO) or Rut (10 and 20 μM) for 20 min and then treated with LTA (10 μg/mL) for 24 h to measure NO (nitrite) by using a Griess reagent. Data are presented as the means ± SEM (*n* = 4). * *p* < 0.05, compared with the control group; ^#^ *p* < 0.05, ^##^ *p* < 0.01, and ^###^ *p* < 0.001, compared with the LTA group.

Although non-steroidal anti-inflammatory drugs (NSAID) have been used to treat joint and spine-related inflammatory pain, but still they create serious side effects including gastrointestinal bleeding, gastritis, ulceration, hemorrhage, and even death [12]. Due to the severe side effects of synthetic pharmaceutical drugs, researchers have paid more attention to searching for nutraceutical compounds as an alternative therapeutic and preventive approach. Nutraceuticals can be delivered in the form of dietary supplements or functional food, supplying beneficial effects in addition to the essential nutritional l components. A recent study has shown the beneficial effects of nutraceutical ingredients on boosting immune functions [13], such as enhancing the infection response mechanism, boosting immunomodulatory activity, and contributing to reducing the impacts of autoimmune disorders and hypersensitivity. Nutraceuticals have also been shown to exert lipid-lowering, anti-inflammatory, anticancer, and antioxidant activity [13,14]. Herbs and other natural plant resources have been regarded as the major sources of several drugs. For thousands of decades, in almost all regions of the world, herbal medicines have been used for treating diseases and disorders. Various herbal formulas are appealed to be effective in treating diseases, however, in most cases the active components in these herbal combinations are indefinite and the mechanisms of action are ambiguous. Therefore, researchers devotedly work in the field of the identification of new components with biological activities. Rutaecarpine (Rut) is a venerable example of a biologically active constituent from natural resources. Rutaecarpine (Figure 1A, 8,13-dihydroindolo-[2’,3’:3,4]-pyrido [2,1-b] quinazolin -5(7H)-one), is one of the fascinating indolopyridoquinazoline alkaloids isolated from *Evodia rutaecarpa.* Studies have demonstrated that rutaecarpine own broad biological and pharmacological properties, such as diuresis, perspiration, uterotonic action, improvement of cerebral functions, antinociception, inhibition of potassium cyanide (KCN)-induced anoxia, specific 2,3,7,8-tetrachlorodibenzo-p-dioxin binding, thermoregulatory, and anti-obesity [15,16]. Due to the beneficial and therapeutic potential of Rut, our previous study examined the underlying molecular mechanisms for the antiplatelet aggregatory effects of this compound. With these effects, a study showed that Rut has an antinociceptive activity that was advocated to be arbitrated by anti-inflammatory action [17]. Nevertheless, the nature of the anti-inflammatory activity of Rut has not been characterized yet. In this milieu, we have investigated the anti-inflammatory mechanism of Rut in bacterial LTA-stimulated macrophages.

## 2. Results

### 2.1. Rut Did Not Affect Cell Viability and Reduce the Level of NO in LTA-Induced Macrophages

A cell viability assay was performed to know whether the concentrations of rutaecarpine used in this study affected the viability of LTA-induced RAW 264.7 cells. Figure 1B shows that Rut at the concentration range of 10–40 µM, together with 10 µg/mL LTA, did not produce cytotoxicity in RAW 264.7 cells (ctl, 100.0 ± 0.0; LTA-activated macrophages, 97.0 ± 4.1; and Rut at 10 µM, 97.8 ± 4.8; 20 µM, 96.9 ± 5.9; 40 µM, 95.8 ± 5.8; *n* = 4).

As shown in Figure 1C, LTA could dramatically induce inflammatory nitrite (NO) production in a concentration (0–20 µg/mL) dependent manner in comparison to the control cells (LTA at 0 µg/mL, 5.2 ± 1.9; 1 µg/mL, 5.9 ± 1.3; 5 µg/mL, 14.0 ± 1.7; 10 µg/mL, 21.3 ± 1.8; 20 µg/mL, 21.3 ± 2.0; *n* = 4). Thus, all subsequent experiments were conducted using LTA at a concentration of 10 µg/mL. Moreover, in Figure 1D, compared with the LTA-induced cells (18.5 ± 2.2), Rut (10 µM, 12.3 ± 2.1and 20 µM, 11.1 ± 1.6)-treated cells significantly (*p* < 0.05) inhibited the LTA-stimulated level of NO production in RAW cells. This outcome designates that Rut inhibits NO production without causing cytotoxicity in LTA-treated RAW cells. Consequently, we selected the ideal concentrations of 10 and 20 µM Rut for further investigation.

### 2.2. Rut Inhibited the Protein Expression of Proinflammatory Molecules in LTA-Induced RAW Macrophages

**Figure 2:**
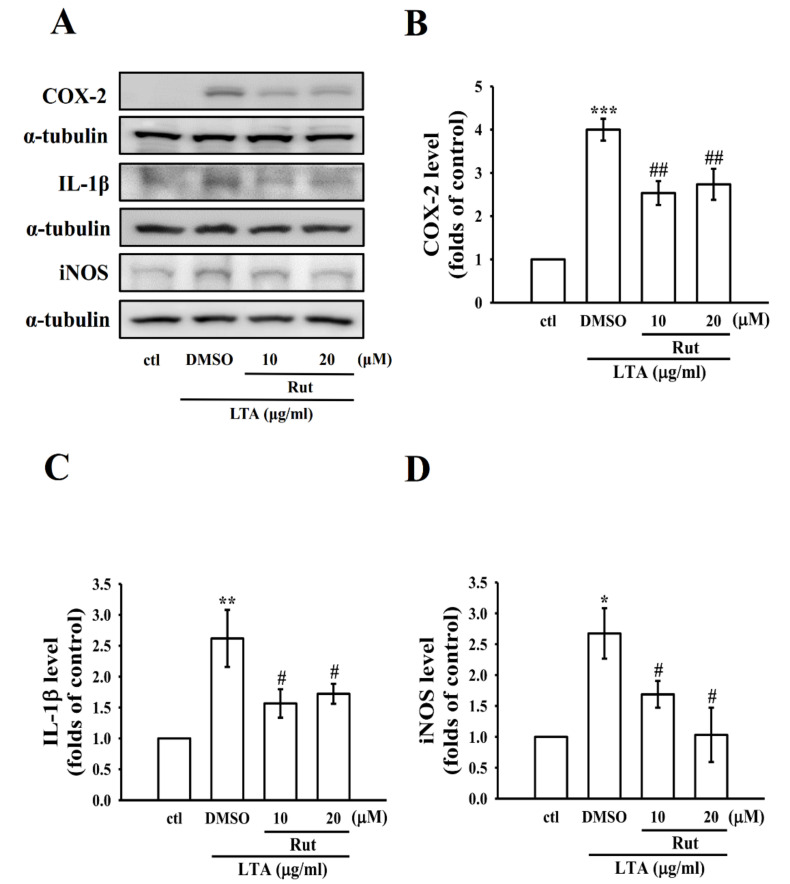
Effects of Rut on the expression of cyclooxygenase-2 (COX-2), interleukin-1 beta (IL-1β), and inducible nitric oxide synthase (iNOS) in LTA-stimulated RAW cells. (**A**–**D**) Cells were pretreated with Rut (10 and 20 μM) for 20 min and then stimulated by LTA (10 μg/mL) for 24 h. The levels of (**B**) COX-2 (**C**) IL-1β and (**D**) iNOS protein expression were evaluated as described in the Methods section. Data are presented as the means ± SEM (*n* = 4); * *p* < 0.05, ** *p* < 0.01, and *** *p* < 0.001, compared with the control group; ^#^ *p* < 0.05 and ^##^ *p* < 0.01, compared with the LTA group.

To explore the properties of Rut on the expression of proinflammatory mediators and cytokines, RAW cells were treated with LTA with or without Rut for 24 h. Elevated expression of COX-2, IL-1β, and inducible nitric oxide synthase (iNOS) in LTA-induced RAW cells was significantly and concentration-dependently reduced by Rut (Figure 2A–D).

### 2.3. LTA-Induced Expression of ERK and p38 but Not c-Jun NH2-Terminal Kinase (JNK) was Inhibited by Rut in RAW Cells

**Figure 3:**
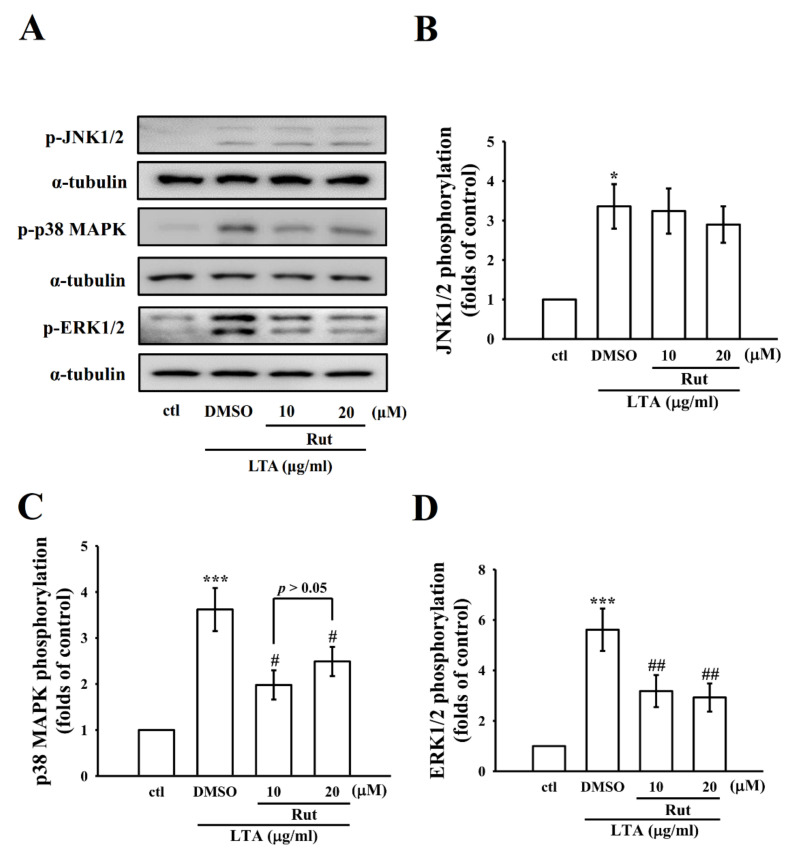
Influence of Rut on LTA-induced phosphorylation of c-Jun NH2-terminal kinase (JNK), p38 mitogen-activated protein kinase (p38 MAPK), and extracellular signal-regulated kinase (ERK) in RAW cells. (**A**–**D**) Cells were treated with 0.1% DMSO or Rut (10 and 20 μM) for 20 min, followed by LTA (10 μg/mL) for 30 min, and the phosphorylation of (**B**) JNK1/2 (**C**) p38 MAPK and (**D**) ERK1/2 were evaluated by the immunoblotting assay as described in the Methods. Data are presented as the means ± SEM (*n* = 4). * *p* < 0.05 and *** *p* < 0.001, compared with the control group; ^#^ *p* < 0.05 and ^##^ *p* < 0.01 compared with the LTA group.

Mitogen-activated protein kinases (MAPKs) are upstream modulators of inflammatory molecules in microglial and macrophage cells. To explore whether Rut inhibits LTA-induced inflammatory events through regulating MAPKs, we observed its effects on LTA-induced MAPK phosphorylation. RAW macrophage cells were pretreated with Rut for 20 min and were then stimulated with LTA for 30 min. Figure 3A–D shows that Rut inhibited LTA-induced ERK and p38 phosphorylation, however, it did not significantly inhibit JNK phosphorylation even at 20 µM concentration. The effects recommend that MAPKs’ signaling pathways, particularly ERK and p38, are involved in Rut’s anti-inflammatory effects in LTA-stimulated macrophages.

### 2.4. Rut Modulates Nuclear Factor Kappa-B (NF-κB) Signaling Pathways to Exert Its Anti-Inflammatory Effects

**Figure 4:**
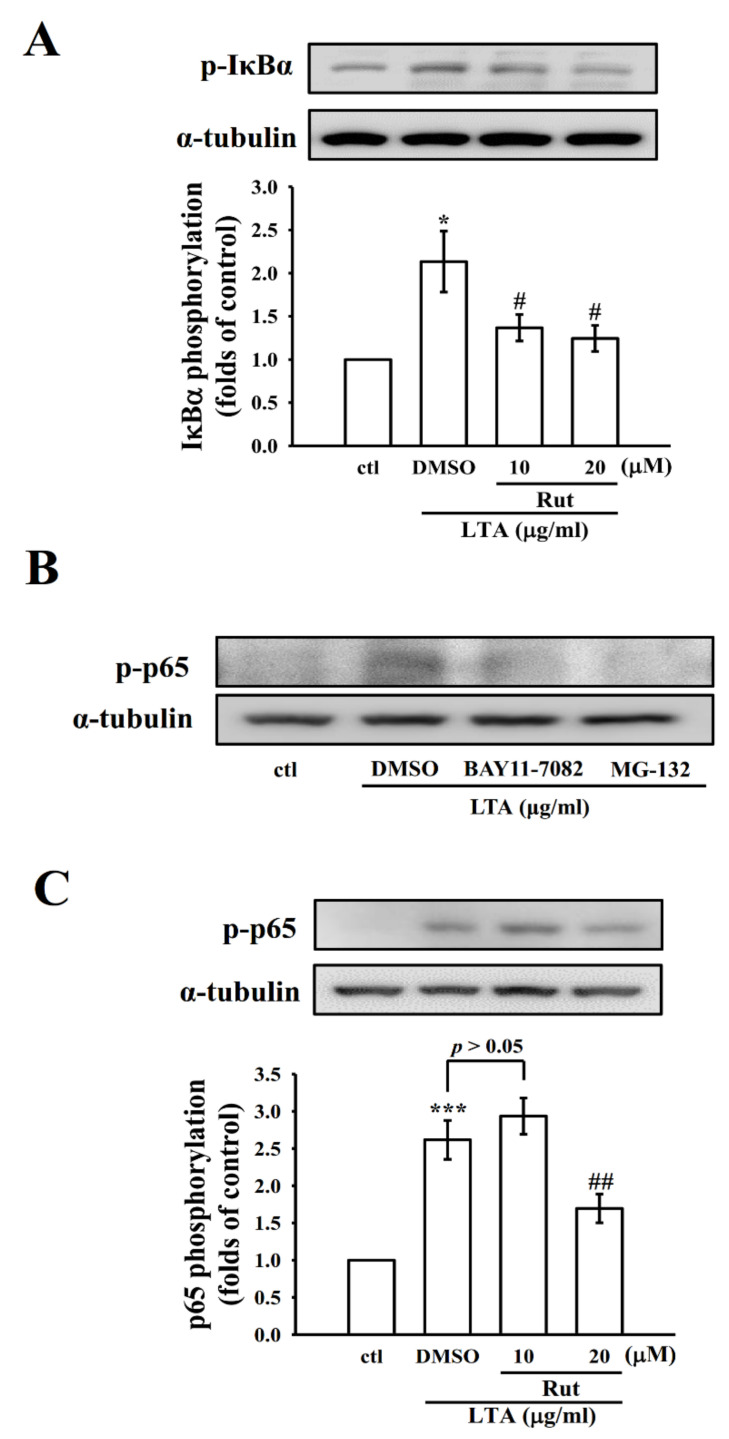
Effects of Rut, BAY11-7082, and MG-132 on IκBα and NF-κB p65 phosphorylation in LTA-stimulated RAW cells. (**A**) Cells were treated with 0.1% DMSO, or Rut (10 and 20 μM) for 20 min, followed by LTA (10 μg/mL) for 30 min. (**B**,**C**) Cells were treated with 0.1% DMSO, BAY11-7082 (10 μM), or MG-132 (50 μM) for 20 min, followed by LTA (10 μg/mL) for 30 min. The phosphorylation of (**A**) IκBα and (**B**,**C**) p65 were determined by immunoblotting. Data are presented as the means ± SEM (*n* = 4). * *p* < 0.05 and *** *p* < 0.001, compared with the control group; ^#^ *p* < 0.05 and ^##^ *p* < 0.01, compared with the LTA group.

It has been stated that inflammatory protein expression upon microglial/macrophage activation is controlled by the transcription factor NF-κB. In the activated macrophages, bacterial endotoxin could activate NF-κB activation, which regulates several inflammatory molecules via releasing NO, iNOS, TNF-α, IL-6, and other inflammatory mediators [18]. In this view, we examined the effect of Rut on the activation of NF-κB in LTA-stimulated macrophage cells. The data showed that LTA induced a distinctive increase in the phosphorylation of inhibitor of nuclear factor κB-α (IκBα) and p65. As shown in Figure 4, Rut pretreatment significantly reduced the levels of p-IκBα and p65 in a concentration (p-IκBα)-dependent manner or at only at high concentration (p65). We also performed control experiments to confirm that pharmacological NF-κB inhibitors BAY11-7082 and MG-132 were effective to affect the NF-κB pathway and it was found that treatment with 10 μM BAY11-7082 or 50 μM MG-132 decreased p-p65 in LTA-induced macrophages (Figure 4B).

**Figure 5:**
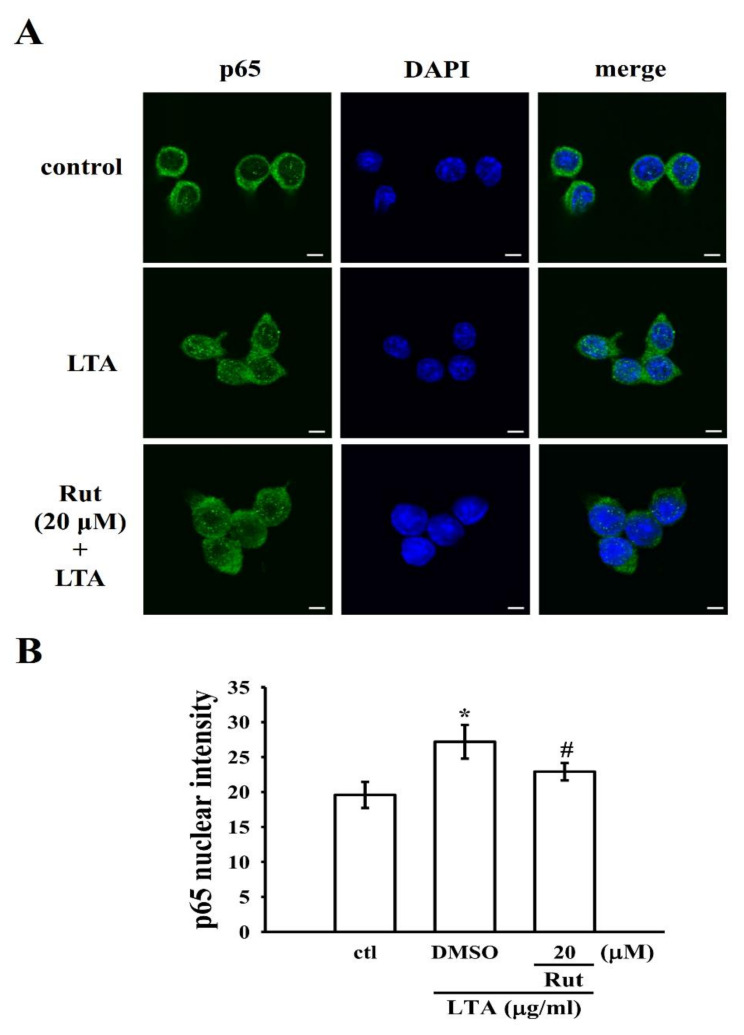
Effects of Rut on LTA-induced NF-κB p65 nuclear translocation in RAW cells. Cells were treated with 0.1% DMSO, Rut (20 μM) for 20 min, followed by LTA (10 μg/mL) for 30 min. (**A**) The immunofluorescence staining analysis was performed with an anti-p65 antibody and FITC-conjugated anti-rabbit IgG antibody (green). 4′,6-diamidino-2-phenylindole (DAPI) was used to label the nuclei (blue). The images were captured by confocal microscopy (scale bar = 5 μm). (**B**) Data were graphed by pooling multiple images, with each individual data point corresponding to the mean fluorescence intensity of each individual cell nucleus. Data are presented as the means ± SEM (*n* = 4). * *p* < 0.05, compared with the control group; ^#^ *p* < 0.05, compared with the LTA group.

**Figure 6:**
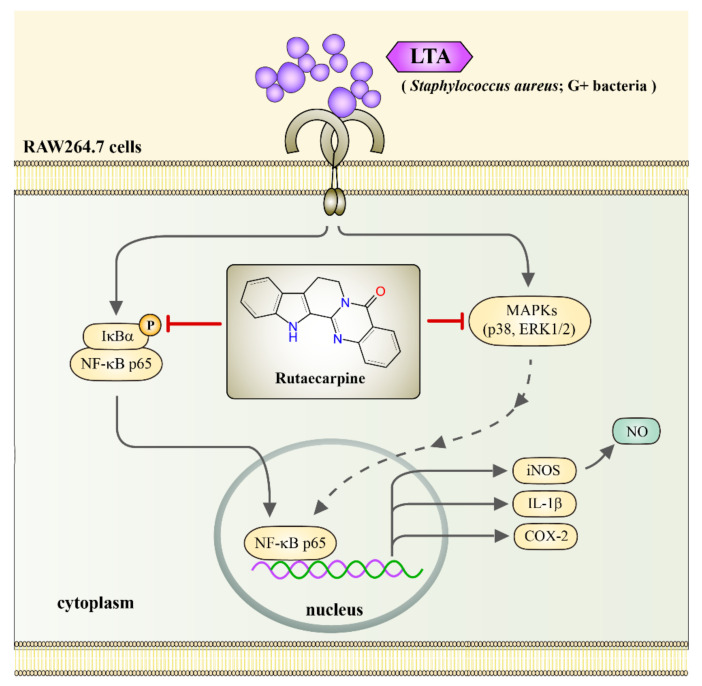
The schematic illustration indicates the inhibitory mechanism of Rut in LTA-stimulated RAW cells. LTA binds to its receptor, induces IκBα/ NF-κB p65 and ERK/p38 phosphorylation, followed by inducing NF-κB p65 nuclear translocation. The translocated NF-κB p65 in turn activates iNOS, IL-1β, and COX-2 expression, iNOS ultimately induces NO production. Rut inhibits LTA-induced production of NO, expression of COX-2, IL-1β, and iNOS, phosphorylation of p38/ ERK1/2, and IκBα/ NF-κB p65, and nuclear translocation of NF-κB p65 in RAW cells, eventually exerts its anti-inflammatory effects.

Dependably, the nuclear translocation of the NF-κB p65 subunit induced by LTA was also reduced by pretreatment with Rut. Briefly, LTA induced FITC-labeled NF-κB p65 (green fluorescence) in the nucleus of RAW cells. However, Rut pretreatment expressively reduced this green fluorescence in the nuclear fraction, which specifies that Rut repressed the nuclear translocation of p65 (Figure 5). Altogether, Rut possibly diminishes the expression of proinflammatory molecules by defeating the nuclear translocation and activation of NF-κB (Figure 6).

## 3. Discussion

Macrophage cells increase the production of proinflammatory molecules after exposure to stimulators such as LPS and LTA via their surface receptors, Toll-like receptor (TLR) 4 and TLR2, respectively. The development of neurodegenerative diseases is reported to be connected with increased expression and activation of TLR2 [19]. Thus, regulating TLR2-mediated microglia/macrophage activation has been recommended as a key therapeutic tactic for treating neurodegenerative diseases.

Rutaecarpine has been shown to own extensive biological and pharmacological properties, such as diuresis, perspiration, uterotonic action, improvement of cerebral functions, antinociception, and anti-obesity [15]. Rutaecarpine displays a notable effect on inhibiting various agonists-induced platelet aggregation [20,21,22]. Recent studies from our group have proven the antiplatelet mechanisms of Rut through suppressing phosphoinositide 3-kinase (PI3K)/Akt/MAPK [23], p38-mediated NF-κB activation [24], and PI3K/Akt/glycogen synthase kinase-3β (GSK3β) [23] signaling cascades. Another relevant study found that Rut diminishes LPS-induced migration by hindering the Src/FAK pathway [25]. In this study, we demonstrated that Rut inhibits inflammatory molecules including NO production, iNOS, COX-2, and IL-1β expression in LTA, a Gram-positive bacterial cell wall component, induced RAW cells. Despite both LTA and LPS interacting with many host factors, they still have different receptors which may differentially control intracellular signaling pathways to induce host immune response. Hence, this study was performed to demonstrate the involvement of Rut in TLR2 (LTA)-induced signaling pathway in RAW cells.

In activated microglia/macrophage, controlling the reactive signaling pathways play a vital role in conserving central nervous system (CNS) homeostasis, as deregulated neuroinflammatory responses can lead to the death of neighboring neurons through the release of inflammatory molecules, such as cytokines, chemokines, NO, and reactive oxygen species (ROS) [26,27]. NO plays a role in the pathogenesis of several inflammatory disorders, and its production in activated macrophages via the rate-limiting enzyme iNOS induces several acute and chronic inflammatory conditions [28]. COX-2 is reported to be overexpressed during the course of LTA-induced inflammatory reaction [29]. Studies have described that the overexpression of iNOS and COX-2 stimulates the activation of NO and PGE2 in activated macrophages, respectively. Overproduction of such inflammatory mediators can result in chronic inflammatory diseases [30]. For instance, endotoxins-induced NO synthesis produces the formation of reactive nitrogen species and neuronal cell death [31]. Inflammatory processes profoundly induce iNOS and COX-2 expression, which increase their products of NO and prostaglandin E2 (PGE2), respectively. IL-1β is believed to be connected to endotoxin-stimulated inflammatory processes [32]. Hence, these proinflammatory molecules might be the prospective targets for any active anti-inflammatory materials. Here, we found that Rut apparently diminishes the production of NO by keeping cells viable in LTA-induced RAW macrophages which were associated with the decreased iNOS protein expression, suggesting that Rut is safe and could be measured as a potential therapeutic agent against inflammatory diseases. A previous study showed that curcumin, the main curcuminoid compound isolated from *Curcuma longa L.* (turmeric) suppressed the secretion of inflammatory mediators TNF-α, NO, and PGE2, and expression of iNOS and COX-2 in BV2 microglia stimulated with LTA [33]. An ethanolic extract of *E. rutaecarpa* inhibits NO production and iNOS expression in LPS-activated microglial and BV2 cells [34]. Xanthotoxin, a furanocoumarin compound was reported to inhibit NO production, and iNOS, COX-2, TNF-α, and IL-β expression in LPS-stimulated RAW macrophages [35]. Correspondingly, similar to LPS-induced cells in other studies, herein Rut has a comparable effect on inhibiting NO, iNOS, COX-2, and IL-β expression in LTA-induced RAW cells. This result indicates that Rut has the ability to inhibit both TLR2- and TLR4-receptors-mediated inflammatory markers.

MAPKs signaling pathways, including ERK, JNK, and p38, participate in the production of a wide variety of neuroinflammatory mediators [36]. ERK is triggered by MAP kinase kinase (MKK) and MKK2, JNK by MKK4 and MKK7, and p38 MAP kinase by MKK3, MKK4, and MKK6 [37]. Triggering of p38 MAPK has been established to be associated with LTA-induced COX-2 expression and PGE2 release in human pulmonary epithelial cells [38]. An earlier study had also exhibited that p38 MAPK activation involved LPS-induced iNOS expression and NO release in RAW cells [39]. LTA-treated RAW cells also induced significant activation of p38 MAPK. SB 203580, a p38 MAPK inhibitor, inhibited LTA-induced iNOS expression and NO release, designating that activation of p38 MAPK is certainly convoluted in LTA-mediated iNOS expression and NO release.

Studies have presented that ERK and JNK activation are vital for NO production [40,41]. Plausible evidence also showed that activation of JNK and ERK plays an important role in tempting the expression of iNOS [42]. A study also found ERK and JNK inhibitors expressively declined the expression of iNOS and production of NO in the microglial cultures [43]. In accordance with the earlier studies, we observed that ERK and JNK were promptly phosphorylated upon LTA exposure. Pretreatment with Rut diminished the phosphorylation of LTA-induced p38 and ERK, however, Rut did not display any suppressive effect on the expression of JNK. This result is fully consistent with the study conducted by Yu et al. [33] as they found that curcumin significantly reduced the phosphorylation of p38 and ERK in LTA-induced BV2 microglial cells, but it was not effective on JNK phosphorylation. These results proposed that Rut instigated the anti-inflammatory effects in LTA-stimulated RAW cells, partially through inhibition of p38 MAPK and ERK activation.

NF-κB plays a vibrant role in NO production in LPS-activated microglia, which is accomplished relatively through the activation of MAPK signaling pathways. In normal cells, the inhibitor of nuclear factor κB-α (IκBα) controls NF-κB by binding and inactivates the transcriptional activity of NF-κB in the cytoplasm. Upon stimulation, an inhibitor of nuclear factor κB kinase β activates that phosphorylates IκBα and results in the degradation of IκBα, consequently releasing NF-κB p50/p65 heterodimer to enter the nucleus. To explore whether the inhibitory effects of Rut on proinflammatory molecules were facilitated by inhibiting the activation of the NF-κB pathway, the phosphorylation of IκBα and NF-κB p65 was examined. The acquired results exhibited that Rut could significantly inhibit the phosphorylation of IκBα and NF-κB p65. The results also indirectly exposed that ERK1/2 and p38 signaling pathways may arbitrate the LTA-induced NF-κB activation and p65 phosphorylation. Altogether, these results direct the vital role of ERK and p38MAPK, but not JNK, in the upstream regulator of NF-κB activation and show a dynamic role in the Rut-mediated anti-inflammatory effects. In accordance with our results, an earlier study found that lucyoside B withdrew the NF-κB transcriptional activity, which was associated with the reduced IκBα phosphorylation, degradation, and the p65 translocation [44]. Another study also found similar results that β-catenin, a member of the WNT/β-catenin signaling pathway, controls the expression levels of NF-κB and inflammatory cytokines in LTA-stimulated BEAS-2B human bronchial epithelial cells [45]. The present study evidently directs that Rut inhibits NF-κB activation and diminishes Gram-positive bacterial toxin-induced inflammation responses. 

## 4. Materials and Methods

### 4.1. Materials

Rut (>98%; Cas no. 84-26-4) was purchased from Cayman Chem (Ann Arbor, MI, USA). Dimethyl sulfoxide (DMSO; Cas no. 67-68-5) and LTA (Cas no. 56411-57-5) were obtained from Sigma (St. Louis, MO, USA). Anti-iNOS (sc-650) polycloncal antibody (pAb) were purchased from Santa Cruz Biotechnology (Dallas, TX, USA). Cell Signaling (Beverly, MA, USA) supplied antibodies for phospho-p38 MAPK Thr180/Tyr182 (9211), phospho-c-JNK (Thr183/Tyr185, 9251), phospho-p44/p42 ERK (Thr202/Tyr204, 9101), phospho-IκBα Ser32/36 (9246), phospho-NF-κB p65 (Ser536, 3033) pAbs. Affinity Biosciences (Cincinnati, OH, USA) provided anti-IL-1β (AF5103) pAb. COX-2 (NB100-689) pAb was purchased from Novus Biologicals (Centennial, Colorado, USA). Fetal bovine serum (FBS, 26140079), Dulbecco’s modified Eagle medium (DMEM; 11965084), L-glutamine penicillin/streptomycin (10378016), and anti-α-tubulin (MS-581-P1) monoclonal antibodies (mAbs) were purchased from Invitrogen (Thermo Fisher Scientific, Waltham, MA, USA). Amersham (Buckinghamshire, UK) supplied horseradish peroxidase (HRP)-conjugated donkey anti-rabbit immunoglobulin G (IgG; RPN4301), and sheep anti-mouse IgG (RPN4201).

### 4.2. Cell Culture and Viability Assay

RAW 264.7 cells were purchased from ATCC (ATCC number: TIB-71). Cells were cultured in DMEM supplemented with 10% FBS and 100 U/mL penicillin G and 100 mg/mL streptomycin at 37 °C in a humidified atmosphere of 5% CO_2_/95% air.

Cells were incubated at a density of 1 × 10^5^ cells per well and then were treated with various concentrations of rutaecarpine (10–40 μM) or solvent control (0.1% DMSO) for 20 min. They were stimulated with LTA (10 μg/mL) or left unstimulated for 24 h. The cytotoxicity of Rut was measured by using an MTT assay. The specified formula of absorbance of treated-cells/absorbance of control cells × 100% was applied to calculate the cell viability index. The absorbance of each well was read at 570 nm using an MRX absorbance reader (Dynex Technologies, Chantilly, VA, USA).

### 4.3. Measurement of Nitric Oxide Concentration

The production of NO synthesis in cell cultures was measured by the Griess method with microplates. To this, cells were treated with Rut (10 and 20 μM) or solvent control (0.1% DMSO) for 20 min and then stimulated with LTA (10 μg/mL) or left unstimulated for 24 h. These conditioned supernatants were collected and mixed with equal volumes of Griess reagent (1% sulphanilamide (Cas no. 63-74-1) and 0.1% naphthalenediamine (Cas no. 1465-25-4) dissolved in 2.5% phosphoric acid). The absorbance of each sample was read at 540 nm by an MRX absorbance reader. Sodium nitrite was used as a standard.

### 4.4. Western Blotting

RAW 264.7 cells in 6-well plates (6 × 10^5^ cells/well) were harvested and lysed in an ice-cold lysis buffer. The lysed or extracted total protein (50 μg) in each sample was separated by 12% sodium dodecyl sulphate-polyacrylamide gel electrophoresis (SDS-PAGE) and transferred onto PVDF membranes (0.45 μm). Following blocking with 5% skimmed milk in TBST buffer (10 mM Tris-base, 100 mM NaCl and 0.01% Tween 20) for 30 min, the membranes were incubated with various targeted primary antibodies (1:1000) for 2 h. This was followed by incubation with HRP-conjugated donkey anti-rabbit IgG or sheep anti-mouse IgG at room temperature for 1 h. The protein band density was achieved by the Biolight Windows Application, V2000.01 (Bio-Profil, Vilber Lourmat, France).

### 4.5. Immunofluorescence Staining Assay

Cells at a density of 5 × 10^4^ cells per well were cultured on coverslips in 6-well plates and treated with 0.1% DMSO or 20 μM Rut with or without LTA stimulation for 30 min, washed with PBS, and then fixed with 4% paraformaldehyde at room temperature for 10 min. Cells were permeabilized with 0.1% Triton X-100 for 10 min and blocked with 5% BSA for 30 min. After incubation, cells were titrated with primary antibodies (1:100) overnight at 4 °C, consequently washed three times with PBS, and titrated with secondary antibodies at room temperature for 1 h. The samples were stained with 4,6-diamidino-2-phenylindole (DAPI, 30 μM) and mounted using a mounting buffer (Vector Laboratories) on a glass slide. The samples were spotted under a Leica TCS SP5 confocal spectral microscope imaging system using an argon or krypton laser (Mannheim, Germany).

### 4.6. Statistical Analysis

Data are expressed as the means ± standard error (SEM). Each experiment was repeated at least four times. Statistical analysis was performed using a one-way analysis of variance (one-way ANOVA). Significant differences among the group were compared using the Newman–Keuls method. The *p*- values < 0.05 were considered statistically significant.

## 5. Conclusions

This study confirmed that Rut had anti-inflammatory activity in LTA-stimulated RAW cells through inhibiting NF-κB and p38 MAPK/ERK activation, but not JNK activation. This inhibition leads to the strong diminution of proinflammatory mediators of iNOS, NO, and COX-2 and cytokine of IL-1β as shown in Figure 6. Besides, Rut does not have cytotoxic effects in RAW cells at its anti-inflammatory dose. Overall, it can be suggested that Rut may have therapeutic potential for some inflammation-related ailments triggered by Gram-positive bacteria. Moreover, this study may provide a new paradigm in natural drug development against NF-κB and p38 MAPK/ERK-mediated inflammatory diseases.

## Data Availability

All data generated or analyzed during this study are included in this published article.
